# Bias of Selection on Human Copy-Number Variants

**DOI:** 10.1371/journal.pgen.0020020

**Published:** 2006-02-17

**Authors:** Duc-Quang Nguyen, Caleb Webber, Chris P Ponting

**Affiliations:** MRC Functional Genetics Unit, Department of Human Anatomy and Genetics, University of Oxford, Oxford, United Kingdom; Fred Hutchinson Cancer Research Center, United States of America

## Abstract

Although large-scale copy-number variation is an important contributor to conspecific genomic diversity, whether these variants frequently contribute to human phenotype differences remains unknown. If they have few functional consequences, then copy-number variants (CNVs) might be expected both to be distributed uniformly throughout the human genome and to encode genes that are characteristic of the genome as a whole. We find that human CNVs are significantly overrepresented close to telomeres and centromeres and in simple tandem repeat sequences. Additionally, human CNVs were observed to be unusually enriched in those protein-coding genes that have experienced significantly elevated synonymous and nonsynonymous nucleotide substitution rates, estimated between single human and mouse orthologues. CNV genes encode disproportionately large numbers of secreted, olfactory, and immunity proteins, although they contain fewer than expected genes associated with Mendelian disease. Despite mouse CNVs also exhibiting a significant elevation in synonymous substitution rates, in most other respects they do not differ significantly from the genomic background. Nevertheless, they encode proteins that are depleted in olfactory function, and they exhibit significantly decreased amino acid sequence divergence. Natural selection appears to have acted discriminately among human CNV genes. The significant overabundance, within human CNVs, of genes associated with olfaction, immunity, protein secretion, and elevated coding sequence divergence, indicates that a subset may have been retained in the human population due to the adaptive benefit of increased gene dosage. By contrast, the functional characteristics of mouse CNVs either suggest that advantageous gene copies have been depleted during recent selective breeding of laboratory mouse strains or suggest that they were preferentially fixed as a consequence of the larger effective population size of wild mice. It thus appears that CNV differences among mouse strains do not provide an appropriate model for large-scale sequence variations in the human population.

## Synopsis

Until recently, it was thought that most inherited human diversity results from genetic variation at single nucleotide sites. However, recent studies discovered many larger-scale differences, involving the duplication or deletion of thousands of bases. Do these large-scale differences contribute greatly to characteristics of human individuals, or are they of little consequence? For clues to solve this mystery the authors looked to the signatures of adaptive evolution written into the DNA. They reasoned that if large-scale DNA differences are beneficial, they should be enriched in genes, particularly those involved in fighting infection and sensing our environment. The authors discovered such enrichments indicating that some large-scale sequence differences have been advantageous during the last approximately 100,000 y of human history. By contrast, modern laboratory mice exhibit few signs of beneficial large-scale DNA differences, perhaps because advantageous sequences have swept rapidly through their ancestral populations. Some large-scale variations in human genomes thus appear to be a legacy of past evolutionary challenges to our species.

## Introduction

How much do different classes of sequence polymorphisms contribute to human phenotypic variation and disease susceptibility? Traditionally, because they are abundant and easily detectable, single nucleotide polymorphisms (SNPs) have been expected to contribute most. Larger-scale polymorphisms, such as duplications, deletions, translocations, and inversions, are less frequent and thus might be thought to have a lesser effect [1].

However, as techniques have improved for detecting polymorphisms at larger scales, evidence has accumulated that these occur far more frequently than hitherto suspected. Some disease-associated genomic rearrangements, for example, are known to arise at least an order of magnitude more frequently than point mutations in human autosomal dominant traits [1]. Moreover, several hundred regions that are variable in copy number have been identified in both human populations [2–5] and mouse strains [6]. Although whether these large-scale copy-number variants (CNVs) are associated with disease is as yet unknown, their abundance and size imply that they may yet be found to underlie functional variation. Nonetheless, relatively few of the human CNVs detected thus far in independent studies overlap [7], indicating that, although numerous, individual CNVs may occur with low minor allele frequencies in the human population.

Sequence variations are usually not uniformly distributed within genomes. In yeast, SNPs are more frequent towards telomeric chromosomal ends [8], as are segmental duplications [9,10], but not apparently CNVs in human DNA [5]. SNPs also occur more frequently within a sequence that is high in G + C content, that has experienced elevated nucleotide substitution rates, and/or that has been subject to reduced selective constraints [11,12]. Consequently, it appears that SNPs have both arisen by mutation and been purified by natural selection, nonuniformly in the human genome.

The assembled human genome sequence is a composite since it is derived from the DNA of many individuals. For any region there is no guarantee that it presents the major allele found in a human population. Indeed, there are three reasons to suppose that rare large-scale sequence variations such as CNVs are not only present, but are overrepresented, in this reference sequence. First, contributing genomes that have been sequenced across boundaries between adjoining paralogous CNV sequences will be favoured for incorporation in the assembly. Second, clone selection for sequencing was biased towards larger insert clones because of the desirability of constructing a minimal tiling set [13]. As a result, clones containing high copy-number regions would be preferred for sequencing over those containing low copy-number regions. Third, because human CNVs, genome assembly gaps, and segmental duplications frequently coincide [2,3,4,5,14], it is plausible that minor allele sequences might be confounding sequence assembly of these regions. We thus predict that an as-yet-unknown proportion of the 5% of the human genome that is highly sequence similar [3,14–16] represents minor allele frequency CNV sequence. It remains to be determined how this 5% partitions between duplications that have been fixed, and thus are present throughout the human population, and others that are polymorphic and are not fixed.

The presence of large-scale minor allelic variants in the reference human genome sequence complicates both CNV experimental design and CNV data interpretation. For example, virtually identical paralogous human sequences are substantially underrepresented in oligonucleotide arrays, thus diminishing the distinction of their copy-number variations in experiments. Furthermore, hybridisation absences may be interpreted as genomic deletions, whereas instead they arise from assaying for minor allelic variants in the reference sequence.

Some CNVs may have been maintained in a subset of the human population due to selective advantage [17], particularly those present at relatively high minor allele frequency. For example, unusually high copy numbers of the *CCL3L1* and *CYP2D6* genes are associated with decreased susceptibility to HIV/AIDS [18] and increased drug metabolism [19], respectively. However, their frequencies suggest that most CNVs have been subject to purifying selection [3].

The fate of CNVs—either fixation or else loss by purifying selection or drift—has been considered theoretically for many decades [17]. Wright's physiological theory [20] predicts that haploinsufficient genes (i.e., those whose loss-of-function alleles strongly affect the phenotype of heterozygotes) experience enhanced fixation of duplicates resulting from selection for increased dosage. Such genes preferentially encode proteins with signalling roles or with binding, regulatory, and structural functions [21,22]. Selective advantage of duplicates due to gene dosage appears to have occurred, for example, for *CCL3L1* [18] and *CYP2D6* [19].

The neutral theory of molecular evolution [23] predicts that a duplicated gene is more rapidly lost by random genetic drift when it arises within larger populations [24,25]. In very large populations virtually all duplications that are rapidly fixed are thus strongly adaptive. By contrast, very small populations are more heterozygous with larger proportions of neutral, slightly advantageous, or disadvantageous duplicates persisting [24].

We were interested in investigating whether CNVs occur preferentially within particular sites and types of human sequence and whether neutral, purifying, or diversifying selection has acted upon them. Our null hypothesis is that CNVs arise uniformly in a genome and are selectively neutral. In this model we expect CNVs not to be enriched in protein-coding genes or other evolutionary, structural, and functional characteristics. To test the model, we surveyed 13 different properties relating to CNVs and CNV genes of human and mouse, and compared these to their genome-wide distributions. Our study relies on recent surveys of CNVs, in particular those of Sebat et al. [3], Iafrate et al. [2], Tuzun et al. [4], and Sharp et al. [5]. We assume that these CNVs have been sampled uniformly from those present in the human population.

We tested whether CNVs occur more frequently, like synonymous substitutions [26], close to telomeres or to pericentromeres, whether they contain unusually high densities of genes, repeats, or G + C base content. We also examined the relative evolutionary rates of CNV genes and their functions. We find that CNVs occur more frequently towards telomeres and centromeres, are enriched in protein-coding genes and simple tandem repeats, but are not elevated in G + C content. Human CNV genes have experienced elevated synonymous and nonsynonymous nucleotide substitution rates, have a deficit of Mendelian disease genes, and have a surfeit of genes encoding secreted and immunity proteins.

Mouse CNVs, on the other hand, possess significantly fewer of the genes that are overrepresented in human CNVs, although they demonstrate the same significant elevation in synonymous nucleotide substitution rates seen for human CNVs. These results indicate that natural selection has acted nonrandomly upon CNVs. We suggest that the different characteristics of human and mouse CNVs we observe may be consequences of these species' contrasting effective population sizes.

## Results

### CNV Properties Relative to Those for the Human Genome

Known human CNVs are neither significantly overpopulated nor underpopulated in densities of RNA genes, interspersed repeats (either considered together, or short or long interspersed nuclear elements considered separately), CpG islands, or G + C content relative to the whole genome (*p* > 0.05). The apparent lack of bias of interspersed repeats and G + C content within CNVs, relative to the remainder of the genome, argues that our conclusions (below) should not be adversely affected by sequence-dependent variations in hybridisation signals [27]. Tissue-specific genes (see Materials and Methods) are also not significantly (*p* > 0.05) over- or underrepresented in CNVs, and no single tissue possessed unusually high or low numbers of CNV genes expressed in that tissue.

By way of contrast, several properties of CNVs are significantly different (*p* < 0.05) from the genome as a whole (Table 1).

**Table 1:**
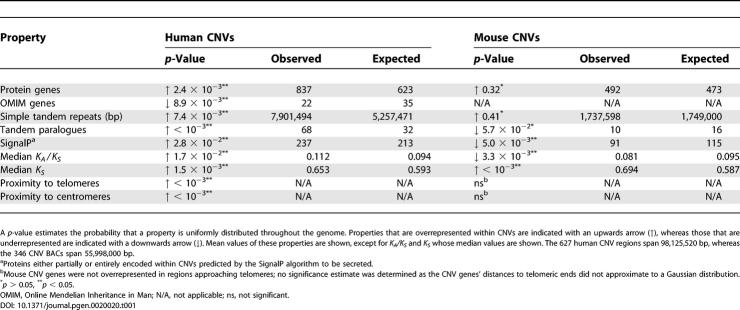
Significance Estimates of CNV Gene Properties

First, human CNVs are significantly overrepresented in number within 2 Mb of telomeres and centromeres (*p* < 10^−5^). By comparing the distributions of CNV distances, either to chromosomal ends or to centromeres, with randomised distributions, we found that regions proximal to telomeres and centromeres contain significantly more CNVs than expected by chance (Figure 1). This observation contrasts with a previous report that these regions are not overrepresented in CNVs [5].

**Figure 1:**
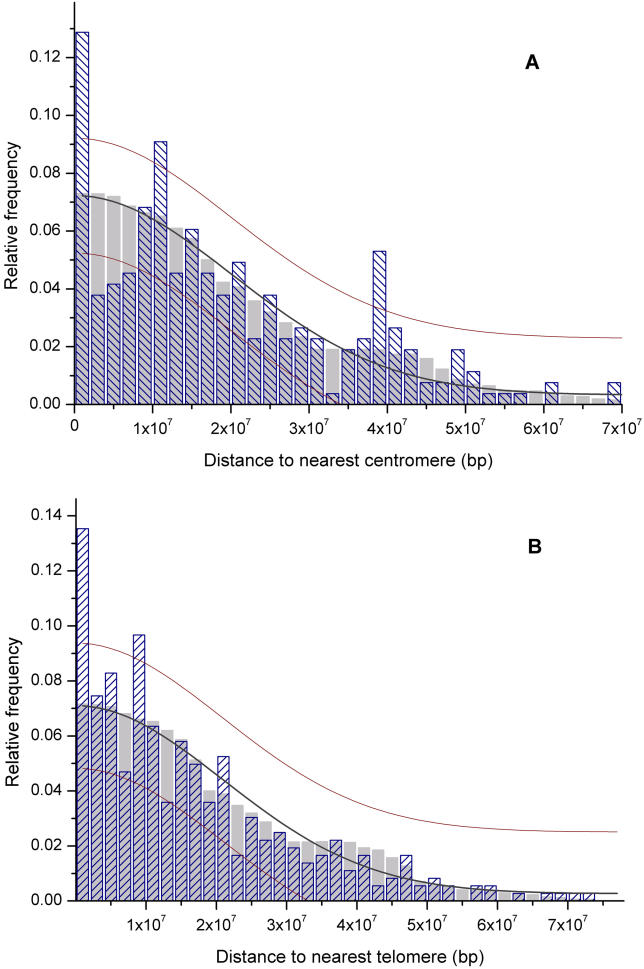
Relative Frequency Histograms of Distances from Human CNVs to the Nearest Centromere or Telomere Relative frequency histograms (striped blue bars) are compared to their expected distributions if CNVs were distributed randomly within the genome (grey bars); these expected distributions are fitted to Gaussian distributions (grey lines). Red lines represent 99.9999% prediction confidence intervals from the fitted curves.

Second, we found that the rates of synonymous substitution (*K_S_* values) for genes within CNVs (median *K_S_* = 0.653) are significantly higher (*p* = 1.5 × 10^−3^) than those for non-CNV genes (median *K_S_* = 0.593). As *K_S_* values are known to be elevated in regions approaching telomeres [26], which are also overrepresented in CNVs (this report), we considered that these two observations might be causally connected. Nevertheless, the significant elevation in *K_S_* persisted even when CNVs within 2 Mb from a telomeric end were discounted (*p* = 1.6 × 10^−2^). We could also discount that high *K_S_* values in CNVs are associated with high G + C or CpG content, since each of these quantities was not significantly different from the genome as a whole (see above).

Third, simple tandem repeats [28], which include microsatellites, but not other repeat types, were also found to be significantly enriched within human CNVs (*p* < 7.4 × 10^−3^). This enrichment is specific to CNVs within 2 Mb of telomeres and centromeres, because when such CNVs were discounted simple tandem repeats were significantly underrepresented (*p* = 0.04).

### Bias of Selection within Human CNV Genes

Human CNVs are also significantly enriched in genes. Those studied here contain 837 complete Ensembl genes. This number is a third higher than expected since, on average, only 624 complete genes were found in each of 10,000 sets of nonoverlapping fragments randomly selected from the human genome, each identical to the CNV set in size distribution. It is also a significantly elevated number since in only 0.24% of randomisations were the gene counts greater than or equal to that of the CNV set (i.e., *p* = 2.4 × 10^−3^). Tandem duplications occur frequently in the mosaic reference human genome assembly [14], and a subset of these may be polymorphic in copy number. Thus, it was not surprising that human CNVs are also significantly enriched in paralogous genes (*p* < 0.001).

Not all gene types, however, are overabundant within CNVs. Genes that are both associated with Mendelian disease and completely contained within human CNVs are significantly underrepresented (*p* = 8.9 × 10^−3^). Such a surfeit could have arisen if null alleles of haploinsufficient genes were more frequently compensated by sequence-similar paralogues, and thus more rarely result in pathology than other genes. This hypothesis predicts that CNV sequences have been purified of fewer mutations than elsewhere in the genome. We do indeed find that SNPs are significantly overrepresented within human CNVs (*p* < 0.001). However, this enrichment may in part be due to an ascertainment bias resulting from difficulties in disambiguating allelic variants (polymorphisms) from close paralogues' sequence differences (*cis*-morphisms) [29].

Using Gene Ontology [30] terms, we also determined that genes involved in acquired immunity, innate immunity, or olfaction are significantly overrepresented (*p* < 0.001) within human CNVs, along with genes encoding integral membrane proteins. Genes encoding intracellular proteins are significantly underrepresented (see Table 2).

**Table 2:**
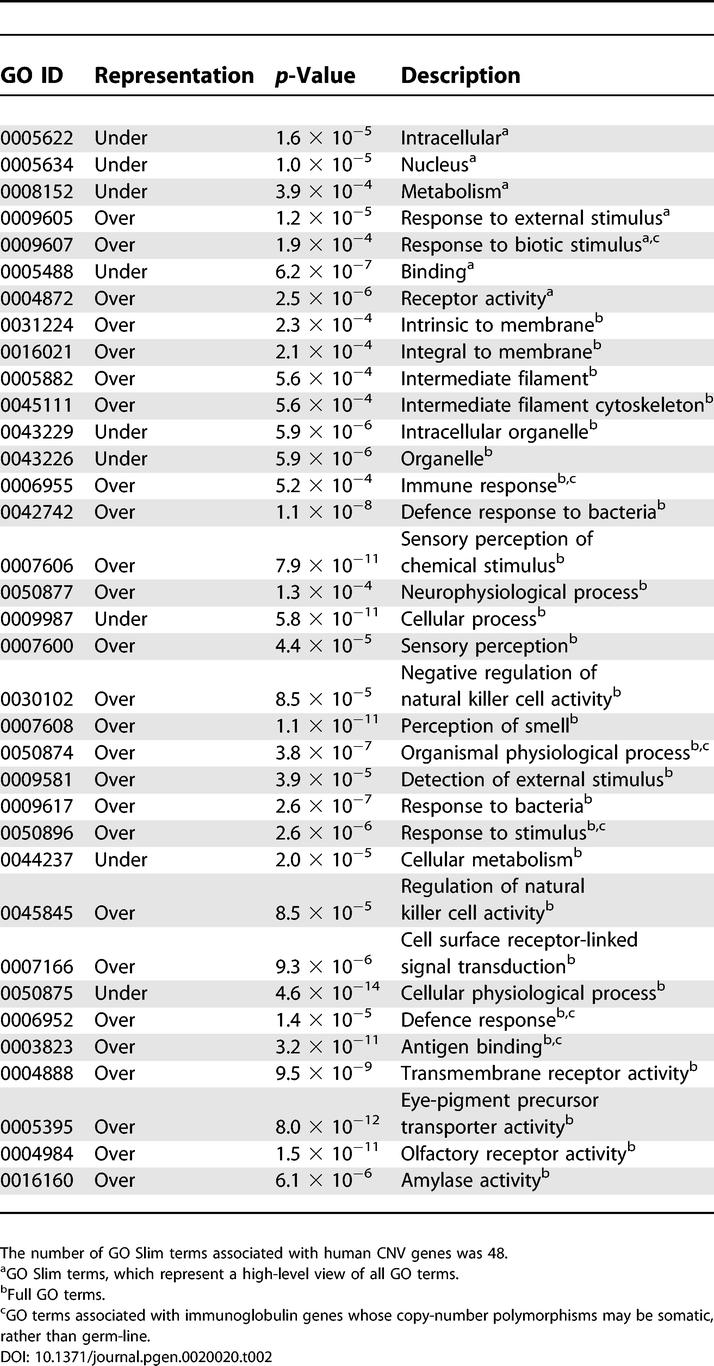
Statistically Significant (*p* < 10^−3^) Over- or Under-Representation of Gene Ontology (GO) Categories in Human CNVs

These findings broadly correspond with expectations from Wright's physiological theory [20] that duplications of haploinsufficient genes improve fitness through selection on increased dosage effects. Haploinsufficient genes are known to be more likely involved in cellular regulation and structure, signal transduction, and various binding functions than are haplosufficient genes [22]. Notwithstanding the underrepresentation of binding proteins, it is notable that several GO terms relating to these functions (for example, intermediate filament, signal transduction, and transmembrane receptors) are overrepresented among CNV genes.

Previous comparisons of mammalian sequences indicate that genes whose functional categories we find to be overrepresented in CNVs (Table 2) frequently have duplicated and/or evolved adaptively, due to competition between individuals or between host and parasite or pathogen [12,31,32]. We can interpret these results (see Discussion) as being consistent with positive selection having acted on some CNV genes within the history of modern humans (approximately last 100,000 y). If so, we might expect CNV genes, on average, to have also accumulated an unusually high number of amino acid-changing (nonsynonymous) substitutions compared with silent (synonymous) substitutions over a much longer time period, the 75–100 million y that separate the mouse and human from their last common ancestor. In other words, they should exhibit an elevation in the average *K_A_/K_S_* ratio—the number of nonsynonymous substitutions per nonsynonymous site *(K_A_)* relative to the number of synonymous substitutions per synonymous site *(K_S_)* [33]—calculated between human and mouse 1:1 orthologues. (Note that only 1:1 orthologues were analysed in order to ensure that lineage-specific paralogues, which often increase their evolutionary rates following duplication [34], do not contribute to the *K_A_/K_S_* distribution.) Indeed, this is the case. Human CNV genes possess, on average, significantly (*p =* 1.7 × 10^−2^) higher *K_A_/K_S_* ratios than those of all 1:1 orthologue pairs (Figure 2). This finding demonstrates that a typical human CNV gene product and its mouse 1:1 orthologue have, on average, diverged unusually rapidly since their common ancestor.

**Figure 2:**
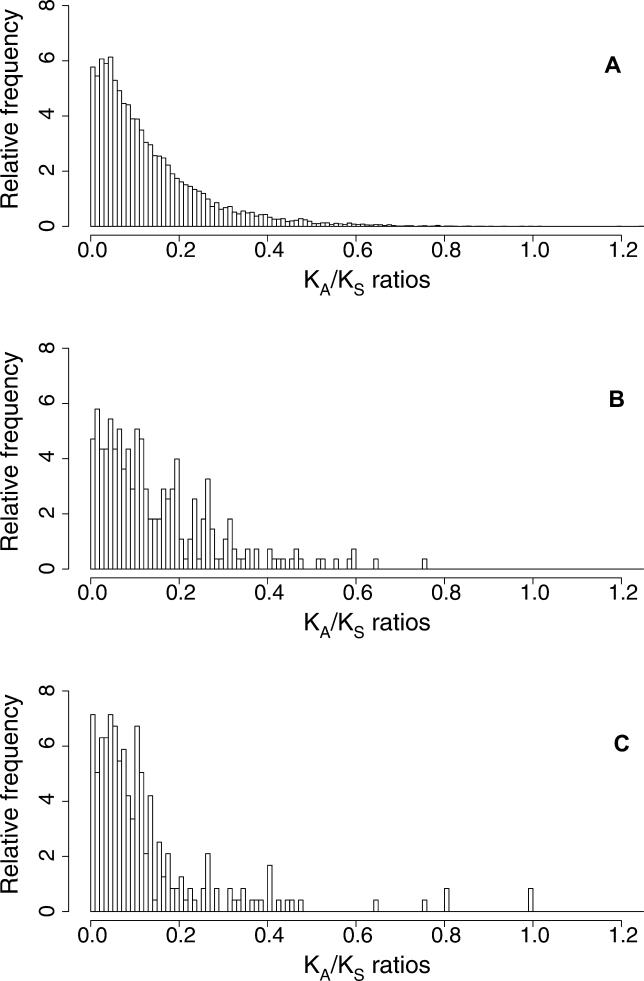
Relative Frequencies of the Ratio of *K_A_* to *K_S_* for Human–Mouse 1:1 Orthologous Genes (A) *K_A_/K_S_* ratios for all human–mouse orthologue pairs (median *K_A_/K_S_ =* 0.094). (B) *K_A_/K_S_* ratios for orthologue pairs of human genes that are completely encompassed in human CNVs (median *K_A_/K_S_ =* 0.112). (C) *K_A_/K_S_* ratios for orthologue pairs of mouse genes completely encompassed in mouse CNVs (median *K_A_/K_S_ =* 0.081). A Kolmogorov-Smirnov test between (A) and (B) demonstrates that *K_A_/K_S_* values are significantly higher, on average, for human genes completely encompassed in human CNVs than for all human–mouse orthologue pairs (*p* = 1.7 × 10^−2^). On the other hand, genes completely encompassed in mouse CNVs exhibit significantly lower *K_A_/K_S_* values than all human–mouse orthologue pairs (*p* = 3.3 × 10^−3^).

In addition to adaptive evolution, *K_A_/K_S_* ratio elevations could also have arisen from recent relaxation of constraints for many genes. However, the only gene family to have suffered numerous and extensive disruptions of coding sequence during primate evolution is the olfactory receptor gene family [14,35]. When such genes are discarded from our CNV gene dataset, the *K_A_/K_S_* ratio elevation remains significant (*p* = 1.5 × 10^−2^). It is thus likely that the *K_A_/K_S_* ratio elevation for CNV genes indicates that they have experienced an unusually large number of adaptive evolutionary events in the past 75–100 million y. This conclusion is consistent with previous reports that segmental duplications contain rapidly evolving gene duplicates [14,15].

### Frequencies of Observed CNVs. Gains and Losses

CNV alleles that are beneficial to human individuals should be segregating at higher frequencies in the general population than neutral CNV alleles, and thus should be observed in a greater number of studies. To examine this expectation, we partitioned our CNVs into those that have been observed in two or more studies and those that have been observed once only. We found that CNVs observed in multiple studies exhibited significantly higher protein-coding genes and simple repeat densities, and higher *K_A_/K_S_* values, on average (Table 3). By way of contrast, CNVs observed in only one study (86% of the total) exhibited none of these significant biases (Table 3). These results are consistent with high-frequency CNVs being preferentially retained in the human population due to their adaptive benefit. We also note that if, as might be expected, the set of rarer CNVs contains a greater proportion of misassignments (experimental errors), then the biases in CNV properties summarised in Table 1 will have been underestimated.

**Table 3:**
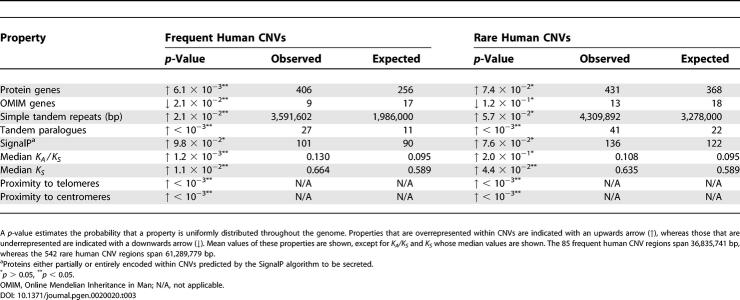
Significance Estimates of Properties of “Frequent” Human CNVs Observed in Multiple Studies or “Rare” Human CNVs Observed in Single Studies

We also partitioned our human CNV set into those involving duplications (“gains”) or deletions (“losses”). (As discussed in the Introduction, some of the high-frequency-loss CNVs will instead represent major, rather than minor, alleles and, thus, will not be true deletions.) We find a significant deficit of Online Mendelian Inheritance in Man disease genes among human gain CNVs but not among loss CNVs (Table 4) as expected if sequence-similar paralogues frequently functionally compensate for null alleles (see above). This deficit may, in part, be due to reduced statistical power to detect significant differences. We also find that loss CNVs do not, on average, possess elevated *K_A_/K_S_* values between 1:1 orthologues (Table 4), which is consistent with duplication, and not deletion, events having provided the substrates of positive selection.

**Table 4:**
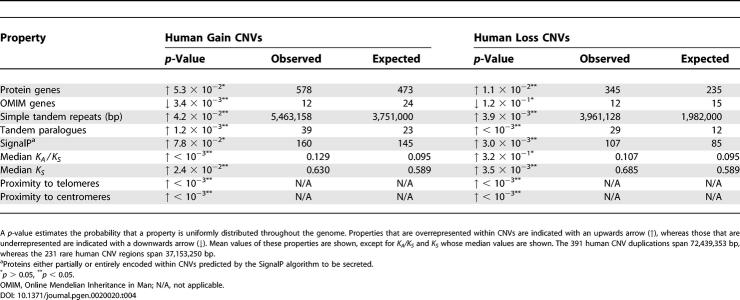
Significance Estimates of Properties of Human CNVs Duplicated or Deleted with Respect to the Human Genome Reference Sequence

### Analyses of Mouse CNVs

We obtained 346 bacterial artificial chromosomes (BACs) containing CNVs among inbred mouse strains [6] that were mapped to 56 Mb of the mouse genome assembly (National Center for Biotechnology Information Build 30). These data presented us with the first opportunity to compare the sequence, evolution, and function of CNV genes in two mammalian species. Strikingly, the only quantity that differed significantly from the genomic background in each of the two species was *K_S_,* calculated between mouse and human 1:1 orthologues (Table 1).

Relative to human CNVs, we find that the set of mouse CNVs analysed better characterises the null hypothesis of random distributions both in the genome and among genes. Mouse CNVs are not significantly enriched in protein-coding genes, paralogous genes, simple tandem repeats, G + C content, or tissue-specific genes (*p* > 0.05) (Table 1). They also exhibit no significant overrepresentation close to telomeres, although this may reflect reduced coverage of BACs in these regions.

Nevertheless, the genes encoded in mouse CNVs, and their associated functions, are strikingly different from those in human CNVs. In only three instances did human and mouse 1:1 orthologues overlap known CNV regions from both species. This finding is unexpected, since the probability of finding this number of 1:1 orthologues, or fewer, in both human and mouse CNVs is 4 × 10^−3^. (This probability was calculated using the hypergeometric distribution using the observations that among approximately 13,000 human:mouse 1:1 orthologues, 418 overlap human CNVs, and 340 overlap mouse CNVs.)

As described above, human CNV genes are enriched in paralogous clusters of the reference genome assembly, they possess elevated *K_A_/K_S_* values, and they encode signal peptide-containing secreted proteins. However, exactly the opposite is true for mouse CNV genes: they are typically not overrepresented in paralogous clusters, they possess significantly decreased *K_A_/K_S_* values, and they are significantly enriched in proteins that lack signal peptides (Table 1). Moreover, in contrast to human CNVs, for which olfactory receptor genes are overrepresented, in mouse CNVs we find these genes to be underrepresented (Table 5).

**Table 5:**
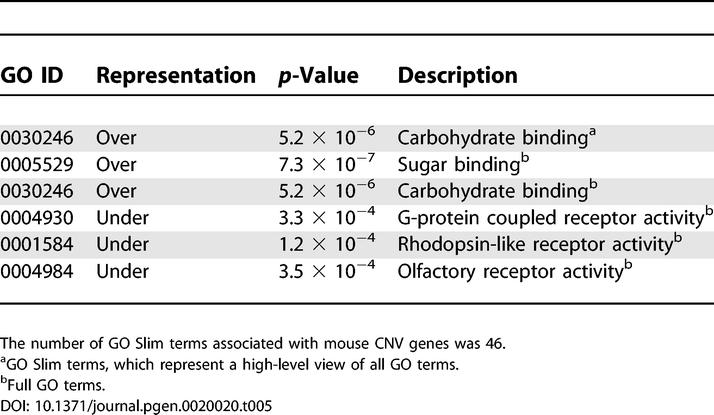
Statistically Significant (*p* < 10^−3^) Over- or Under-Representation of Gene Ontology (GO) Categories in Mouse CNVs

Only carbohydrate-binding genes are significantly (*p* < 0.001) overrepresented in mouse CNV BACs. This enrichment is almost entirely due to natural killer cell lectin-like receptor *Ly-49* paralogues [36]. Sequence variations between different mouse strains have been shown to influence ligand-binding affinities [37]. Rather than being allelic variants, as reported previously [37], these sequence variants may thus instead represent distinct paralogues that have segregated differentially, as CNVs, among mouse strains since their common origin.

## Discussion

Our results are relevant to three key issues of CNV evolution: the mutational variation of polymorphic duplication, the contribution of CNVs to phenotypic diversity and disease, and the differences in large-scale sequence variation between two distinct mammalian species. Each of these three issues now will be discussed in turn.

### Mutational Variation of CNVs

Both human CNV and mouse CNV sequences appear to be unusually susceptible to synonymous nucleotide substitutions. We assume that the nonuniform genome-wide distribution of CNVs is due, at least in part, to variable segmental duplication rates. Indeed, duplicates can themselves seed further duplication events by nonallelic homologous recombination [38]. It thus appears that segmental duplication and nucleotide substitution mutational rates are regionally correlated. Since G + C levels and synonymous substitution, neutral, and recombination rates all strongly and positively covary [11], we might expect CNV sequences to be typically associated with high levels of recombination and G + C content [11,39]. Nonetheless, neither human nor mouse CNVs possess atypical G + C compositions, and human CNVs are overrepresented in pericentromeric sequences, when these are usually characterised by suppressed, rather than elevated, recombination rates [39]. Notwithstanding the higher densities of human CNVs close to telomeres and centromeres, and in repetitive and high *K_S_* regions (Table 1), we find no single factor that might explain their chromosomal distributions.

### Adaptation, Phenotypic Variation, and Disease

Our results indicate that a subset of human CNVs, particularly those found at high minor allele frequency, has been retained in the human population as a result of positive selection. We found that human proteins encoded within CNVs possess, on average, unusually high *K_A_/K_S_* values (measured against their single mouse orthologues) that is consistent with a proportion of these genes having evolved adaptively. It is notable that genes that have evolved the most rapidly or have duplicated, when mammalian sequences are compared [12,31,32], often correspond to those that are most overrepresented in human CNVs. Human CNV genes possess significant enrichments in chemosensation and immune response functions (Table 2), which have well-documented roles among mammals in adaptation to novel environmental niches [31,32]. Indeed, it is only these two functions that greatly contribute to the CNV gene *K_A_/K_S_* elevation because when their associated genes (namely, those encoding olfactory receptors, β-defensins, and immunoglobulins) are discarded, no significant difference in *K_A_/K_S_* values is then observed.

Increased protein sequence divergence is also reflected in the enrichment of paralogous genes and signal peptide-encoding genes in human CNVs (Table 1) since each of these categories is associated with increased protein sequence divergence in mouse–human comparisons [12,31]. Our observation that human CNVs encode unusually high numbers of genes may also be attributed to positive selection. We discount an alternative hypothesis that the gene richness of CNVs is associated with an elevated G + C because we found no significant differences between the G + C content distributions of human or mouse CNVs and those of their genome assemblies (*p* = 0.28 and 0.26, respectively). Instead, the elevated gene density of CNVs may have arisen because of the retention of duplicated sequences that were of adaptive benefit and the purification by selection or drift of those that were not.

The overabundance of immunity and chemosensation genes in human CNVs implies that they might have been selectively favoured in recent evolution. Indeed, selection on gene copy number is reported for *CCL3L1,* an immune response gene, where relatively low copy number is associated with increased susceptibility to HIV/AIDS [18], and it remains possible that copy-number variation of olfactory receptor genes underlies individuals' sensitivities to specific odorants [40,41].

An alternative hypothesis is that the unusual abundance of “environmental genes” within human CNVs results from adaptation that occurred not during recent hominin evolution, as we have just proposed, but instead from earlier in the primate lineage. In this scenario, such genes are enriched in human CNVs simply because their forebears' duplications generated repetitive sequences that then have preferentially seeded tandem duplication and CNVs by nonallelic homologous recombination. This issue remains unresolved owing to difficulties in distinguishing mutational biases from selective biases. Nevertheless, it would be curious if adaptive episodes that occurred earlier in the primate lineage (and elsewhere within the mammalian clade [12,42]) were to have discontinued only in recent times. Moreover, in this study we found no evidence that other repetitive sequences—namely, human interspersed elements and mouse tandem paralogues—have preferentially seeded CNV duplications. Consequently, we believe it more likely that the biases in human CNV properties we observe are mainly due to adaptive events in the last 100,000 y of human history.

We found that there is a significant deficit of Mendelian disease genes within human CNVs. From one perspective, rather than this deficit, a surfeit might be expected. This is because such genes in general are overrepresented in rapidly mutating (high *K_S_*) sequence [43,44]. Nevertheless, despite CNV sequences experiencing unusually rapid synonymous substitution rates (see above), they contain significantly fewer Mendelian disease genes than expected. The disease gene deficit may thus be due in part to functional compensation afforded by CNV paralogues.

Moreover, because tandemly repeated sequences, such as microsatellites and paralogous genes, are a potent substrate for human genomic rearrangement via nonallelic homologous recombination [38], CNVs might be thought to promote disease-associated mutations. Although such events may occur, CNVs may also buffer the genome against deleterious mutations if their paralogous, essentially identical, genes compensate for one another [45]. Gene compensation, together with the frequent lack of account taken of polymorphic sequence-similar paralogues when candidate disease genes are sequenced, may help to explain the underrepresentation of Mendelian disease genes in CNV regions.

### The Effect of Population Size on the Rate of Fixation of CNVs

Mouse CNV genes differ from their human counterparts in possessing significantly lower than average *K_A_/K_S_* values, and lower fractions of signal peptide-encoding genes (Table 1). Moreover, the number of orthologue pairs that are present in both human CNVs and mouse CNVs is unexpectedly low, and there are no functional categories that are overrepresented in both species' CNVs (Tables 2 and 5).

One explanation for these observations might be that selection itself has acted on very different human and mouse genes. This interpretation appears unlikely since selective constraints on gene functions are strongly correlated when these are compared between murids and between hominids [46]. Other explanations might be that these results are artifacts, arising from the different technologies and samples used in identifying CNVs in the human population and among mouse strains, or that the 2.4-fold fewer mouse CNVs than human CNVs in this study results in a reduced power to detect significant deviations. Although these remain possibilities, the finding that synonymous substitutions are significantly overrepresented in CNVs from both species and that *K_A_/K_S_* values and signal peptide-encoding genes (Table 1) are significantly lower among mouse CNV genes appear to argue against these. A further explanation might be that selective breeding during the recent generation of laboratory mouse strains led to “adaptive” CNV gene duplicates (such as olfactory receptor genes and genes encoding secreted proteins) unwittingly being purged preferentially from these lines. This final possibility will need to be investigated by surveying CNVs from wild mice populations.

Finally, the differences between human and mouse CNV properties may be explained if advantageous duplications were fixed in the mouse more frequently than they were in humans. According to the nearly neutral theory of molecular evolution, mildly deleterious, neutral, or advantageous duplicates persist for longer, on average, in smaller populations than they do in larger populations [24,47]. For very large effective population sizes, virtually the only gene duplications that are fixed are those that are strongly advantageous. The effective size of the modern human population (approximately 10^4^ [48]) is up to two orders of magnitude smaller than that for the house mouse Mus musculus (approximately 5 × 10^5^ to 8 × 10^5^ [49]). Furthermore, different laboratory mouse strains still exhibit many of the sequence variations expected to separate these strains' three founder subspecies, M. musculus subsp. *musculus, M. musculus* subsp. *domesticus,* and M. musculus subsp. *castaneus* [50], indicating that, collectively, the effective population size of laboratory mouse strains should not be greatly reduced from that of M. musculus in the wild.

Over equivalent numbers of generations, we expect the mouse population thus to have fixed more advantageous, and purified more disadvantageous, mutations than the human population. As a consequence, fewer advantageous duplications will remain as polymorphisms among extant mouse strains compared to the human population.

This model predicts a decrease in average *K_A_/K_S_* values for mouse CNV genes, when compared with their 1:1 human orthologues, consistent with that seen in Figure 2. This is because duplicated “adaptive” genes (such as those encoding olfactory receptors and secreted proteins [31]; see Table 2) often exhibit unusually elevated sequence divergence, and when these are fixed in the mouse population they then deplete the mouse *K_A_/K_S_* distribution of high values. A consequence of the lower effective population size of humans is that a greater fraction of advantageous duplications will be fixed at essentially the same slow rate as neutral mutations.

Human CNVs are thus expected to encode disproportionately large numbers of proteins that typically contribute most to adaptation, i.e., those that are secreted and that exhibit high sequence divergence between human and mouse [31]. The model thus accounts for both the unusually high average human–mouse *K_A_/K_S_* value for human CNV genes (Figure 2) and their enrichment in genes encoding signal peptides (Table 1).

For the mouse this scenario predicts that genetic drift preferentially has purged deleterious, neutral, and even slightly adaptive duplications, whilst many strongly adaptive duplications have been fixed at significantly increased rates than for the human population. Future investigations of CNVs from other species associated with contrasting effective population sizes should help to clarify the validity of this evolutionary model.

In summary, whereas evidence is scarce that human SNPs have contributed frequently to adaptive evolution [12,46,51], in human CNVs the increased densities of all genes, and in particular “adaptive genes” exhibiting elevated coding sequence divergence, provide evidence of advantageous duplications that have yet to become fixed in the human population.

## Materials and Methods

We obtained 823 human CNVs from the Database of Genomic Variants (http://projects.tcag.ca/variation [version June 2005]) that had been mapped to the human genome assembly (National Center for Biotechnology Information Build 35). These CNVs correspond mainly to those identified by Sebat et al. [3], Iafrate et al. [2], Tuzun et al. [4], and Sharp et al. [5]. Overlapping CNVs were merged, resulting in 627 distinct CNV regions. Among these CNV regions, those identified by two or more independent studies were subclassified as “frequent,” while those observed once were designated as “rare.” CNV regions were also partitioned into those that were duplicated (“gains”) or else deleted (“losses”) on the basis of information reported in the Database of Genomic Variants. It should be noted however that assignment of gain or loss is entirely dependent on the control used for the experiment.

Gene predictions and corresponding Gene Ontology (GO) and GO Slim (http://www.geneontology.org/GO.slims.shtml) terms, signal peptide [52], human disease association (via the Morbid Map subset of the Mendelian Inheritance in Man Database [53]), and protein family annotations were assigned to CNVs according to Ensembl [54] (Ensembl mart version 31). A similar procedure was used for 346 mouse BACs known to be variable in copy number among 14 mouse strains [6] that had been mapped to the mouse genome assembly (National Center for Biotechnology Information Build 30). Gene predictions for genes within these CNVs were obtained from Ensembl (Ensembl mart version 19.1). Single orthologues in human and mouse were taken from a previous study [55]. A total of 13,111 Ensembl mouse genes possessed single orthologues in human, whereas 13,357 Ensembl human genes possessed single mouse orthologues. (The small discrepancy between these orthologue counts arises from gene predictions discarded between different Ensembl versions.) Genes were considered paralogous if they possessed the same Ensembl family identifier.

Simple tandem repeats (from Tandem Repeats Finder [28]), SNPs (from dbSNP, http://www.ncbi.nlm.nih.gov/projects/SNP), RNA genes (microRNAs and small nucleolar RNA), CpG islands, G + C content, interspersed repeats, and telomeric or centromeric locations were obtained from the University of California Santa Cruz's genome browser [56] (http://genome.cse.ucsc.edu human: hg17, mouse: mm3). Gene expression data (GNF Expression Atlas 2 data for human [57] and for mouse [58]) were used to define tissue-specific genes (i.e., genes possessing at least a 4-fold-higher expression level in one or more tissues relative to the median expression in all tissues) and also genes highly expressed in particular tissues (i.e., those where the average difference (AD) between sense tags and missense tags exceeds 200). *K_A_* and *K_S_* values and their ratios were calculated for 1:1 orthologues using the yn00 method of Yang and Nielsen [59].

To test the null hypothesis that a property is higher, or lower, in known CNVs than elsewhere in the genome, we performed a randomisation test. For this, 10,000 sets of regions were sampled randomly from the genome assembly; these regions were matched in both number and size to the CNV set. This test assumes that the set of CNVs we considered is representative of all CNVs present in the human population. We calculated the fraction *p* of such randomly chosen regions that contained higher, or lower, values of the property. Values of *p* > 0.05 were considered to indicate that the CNV data were not significantly different from the genome data taken as a whole.

The likelihood that a GO annotation is over- or underrepresented among CNV genes was estimated using the hypergeometric distribution [60]. The probability that two sets of *K_A_, K_S,_* or *K_A_/K_S_* values are sampling an equivalent distribution was calculated using the two-sided Kolmogorov-Smirnov test [61]. The likelihood that CNVs are overrepresented in regions close to telomeres or centromeres was estimated by fitting to a Gaussian distribution (using Origins 7.5 software from OriginLab, Northampton, Massachusetts, United States).

## Abbreviations

BAC: bacterial artificial chromosome
bp: base pair
CNV: copy-number variant
*K_A_*: number of nonsynonymous substitutions per nonsynonymous site
*K_S_*: number of synonymous substitutions per synonymous site
SNP: single nucleotide polymorphism
